# Utility of GnRH Agonists before embryo transfer in women with adenomyosis: Systematic review and meta-analysis

**DOI:** 10.5935/1518-0557.20250012

**Published:** 2025

**Authors:** Mireia González-Comadran, Esteban Alwane Olmos, Mauricio Alexis Agüero Mariño, Miguel Angel Checa Vizcaíno

**Affiliations:** 1 Department of Obstetrics and Gynecology, Hospital del Mar, Barcelona, Spain; 2 Obstetrics and Gynaecology Department, Universitat Autònoma de Barcelona, Cerdanyola del Vallès, Spain; 3 Department of Gynecology and Infertility, Clinica CER Reproducción Asistida, Santiago, Chile; 4 Fertty Clinic, Barcelona, Spain; 5 Faculty of Medicine and Life Sciences, Pompeu Fabra University, Barcelona, Spain

**Keywords:** Adenomyosis, infertility, Ultra-long GnRH agonist protocol, Long GnRH agonist protocol, antagonist protocol, pituitary downregulation.

## Abstract

This systematic review and meta-analysis aimed to evaluate the benefit of GnRH agonist (GnRHa) in reproductive outcomes among women with adenomyosis undergoing IVF. The utility of GnRHa protocols for controlled ovarian stimulation (COS) and fresh embryo transfer, and the pretreatment with GnRHa before frozen-thawed embryo transfer were evaluated. The search spanned studies published in MEDLINE and Embase databases up to April 2024. Eight retrospective studies were included. The use of long GnRHa and antagonist protocol before COS exhibited significantly higher implantation rate compared to ultra-long GnRHa (OR 1.1, 95% CI 0.69-1.77 and OR 1.98, 95% CI 1.04-3.75, respectively), although no significant differences were observed in clinical and live birth rates. However, antagonist compared to long GnRHa protocol before COS improved live birth rate (OR 2.59, 95% CI 1.03-6.52). Pretreatment with GnRHa before FET among women with adenomyosis did not improve reproductive outcomes. In conclusion, there is no evidence regarding benefit of long or ultra-long GnRHa protocol before COS or before FET among women with adenomyosis undergoing IVF. In fact, the use of long GnRHa seem to worsen reproductive outcomes compared to antagonist protocols. Prospective trials are needed to assess the potential benefit of GnRHa among women with adenomyosis seeking fertility.

## INTRODUCTION

Adenomyosis is a benign gynecological disease characterized by the invasion of endometrial glands and/or stroma within the myometrium, leading to hypertrophy and hyperplasia of the surrounding smooth muscle cells (Ferenczy, 1998). According to a population-based cohort study with 333,693 women, the overall incidence was reported to be 1.03% or 28.9 per 10.000 women-years (Yu *et al.*, 2020).

For decades, adenomyosis was considered a disease that affected multiparous women over the age of 40 years. Nowadays, with the widespread use of TVUS (transvaginal ultrasonography) and the implementation of the ultrasonographic features to detect adenomyosis, clinicians are able to diagnose the disease at earlier stages, especially among nulliparous women (Pinzauti *et al.*, 2015; Harada *et al.*, 2016; Tellum *et al.*, 2020).

Adenomyosis is linked to infertility. Even though the rationale behind this relationship remains not clearly established, several authors have reported a negative impact on reproductive outcomes among these women. In a meta-analysis by Vercellini *et al.* (2014), a 28% reduction in the likelihood of clinical pregnancy and an increased risk of miscarriage was reported among women with adenomyosis undergoing IVF compared with women without the disease.

Later Younes & Tulandi (2017) confirmed in another meta-analysis a 41% decrease in live pregnancy rates and an increased risk of miscarriage among women with adenomyosis. Consistently, in two other systematic review and meta-analysis, both Horton *et al.* (2019) and Huang *et al.* (2020) also described an increased risk of miscarriage among these women.

In this regard, the use of long and ultra-long GnRH analogue protocols as pretreatment before COS has been considered of choice by many clinicians in an attempt to mitigate the hyperestrogenism that results from the increased expression of estrogen receptors, and the functional link between estradiol action and adenomyotic cell proliferation (Sztachelska *et al.*, 2022). However, conflicting results have been reported in terms of reproductive outcomes.

In the meta-analysis by (Nirgianakis *et al.*, 2021), women with adenomyosis were associated with a significant reduction in clinical pregnancy are and higher miscarriage rate after IVF, especially when following short GnRH agonist or antagonist protocol for controlled ovarian stimulation (COS). In contrast, Cozzolino *et al.* (2022), found that the use of GnRHa downregulation did not show significant benefits in reproductive outcomes.

The aims of this systematic review and meta-analysis are to (i) evaluate the effect of GnRH agonist protocols for controlled ovarian stimulation, and (ii) determine whether the pretreatment with GnRH agonist before frozen-thawed embryo transfer improve reproductive outcomes among women with adenomyosis.

## MATERIALS AND METHODS

The study was exempt from Institutional Review Board approval because this was a systematic review and meta-analysis. The review was made in accordance with the EQUATOR Reporting Guideline (EQUATOR, 2004). The review protocol was registered in International Prospective Register of Systematic Reviews (PROSPERO) (ID: CRD42024555052).

### Search strategy

The systematic review spanned MEDLINE and Embase databases up to April 2024. The search combined terms and descriptors related to adenomyosis, GnRH agonist, pituitary down regulation and embryo transfer. The search strategy was modified to comply with the requirements of each database consulted. The reference lists of all the relevant articles and overviews was screened to identify additional relevant citations. No language limits were used. The complete search strategy is available upon request from the authors.

### Eligibility criteria

The review included randomized controlled clinical trials and cohort studies of women with adenomyosis diagnosed by ultrasound or magnetic resonance imaging (MRI). The types of interventions evaluated were (i) long compared to ultra-long pituitary downregulation with GnRHa prior to controlled ovarian stimulation (COS), (ii) short, long or ultra-long GnRHa compared to GnRH antagonist protocol before COS, (iii) long or ultra-long GnRHa compared to no GnRHa pretreatment prior to FET regardless of the COS protocol followed.

Long GnRHa protocol was defined as the use of GnRH analogues starting at the mid-luteal phase of the cycle prior to COS, while the ultra-long protocol was defined as the use of GnRHa at least one month prior to COS.

Studies that mixed women undergoing different GnRH agonist protocols within the same group, or that included combined treatments for adenomyosis such as surgery or radiofrequency were also excluded.

### Data extraction and outcome measures

The data were collected using standard forms in which the characteristics of the study design, participants, interventions, comparisons, and main outcomes were recorded (Tables 1 and 2). Where data were missing, the original review authors were contacted for assistance. Two independent authors (E.I.A.O. and M.G.C.) judged study eligibility, assessed the risk of bias and extracted data solving discrepancies by agreement, and if needed, reaching consensus with a third author (M.A.C). The agreement between reviewers was analyzed using the weighted kappa for each inclusion criterion (Fleiss, 1993).

**Table 1:** Description of the interventions by treatment group of the included studies.

Comparison	Study	Intervention group		Control group		Outcomes
		Protocol	Type of ET	Protocol	Type of ET	
**GnRH protocol before COS and fresh ET**		**Ultralong GnRHa protocol**	**Long GnRHa protocol**			
	Hou *et al*., 2020	Triptoreline acetate 3.75mg monthly for 3 months before COS. Cos started 30-45 days after the last GnSRHa injection	Fresh	Triptoreline acetate 0.1mg daily in the mid-luteal phase for 10 days followed by 0.05mg daily until de day of HCG injection. COS was started 10 days after the first GnRHa injection	Fresh	Clinical pregnancy rate, implantation rate, Miscarriage rate, Live birth rate
	Chen *et al*., 2020	Triptoreline acetate 3.75mg monthly. Dose repeated up to 3 months if AP uterine dimameter was ≥ 70mm. Triptoreline acetate depot (1.0-1.8mg) or daily dose (0.05-0.1mg) was used for pituitary downregulation 28 days after the last monthly injection. COS was iniciated 14 days after the last injection for pituitary downregulation	Fresh	Triptoreline acetate 3.75mg monthly. Dose repeated up to 3 months if AP uterine dimameter was ≥ 70mm. Triptoreline acetate depot (1.0-1.8mg) or daily dose (0.05-0.1mg) was used for pituitary downregulation 28 days after the last monthly injection. COS was iniciated 14 days after the last injection for pituitary downregulation	Fresh	Clinical pregnancy, miscarriage and live birth rate
	Lan *et al*., 2021	Triptoreline acetate 3.75 monthly for 2-4 months before COS. COS started 28 days after the last GnRHa injection	Fresh	Triptoreline acetate single dose of 0.93 - 1.87mg in the mid-luteal phase. COS was started 14 days after the GnRHa injection	Fresh	Biochemical pregnancy, implantation, miscarriage, clinical pregnancy, live birth rate
	Wu *et al*., 2022	Triptoreline acetate 3.75mg monthly for 3 months. A 4th inhection was given if AP uterine diameter was ≥ 70mm. COS started 28 days after the last GnRHa injection	Fresh	Triptoreline acetate depot (1.0 + 1.8 mg) or daily dose (0.05 + 0.1mg) for pituitary downregulation. COS started 28 days after the last GnRHa injection	Fresh	Biochemical pregnancy, implantation, miscarriage, clinical pregnancy, live birth rate
	Ge *et al*., 2023	Triptoreline acetate 3.75mg monthly. Dose repeated up to 6 months if AP uterine diameter was ≥ 70mm.	Fresh	Triptoreline acetate 3.75mg monthly. Dose repeated up to 6 months if AP uterine diameter was ≥ 70mm.	Fresh	Biochemical pregnancy, implantation, miscarriage, clinical pregnancy, live birth rate
		**Ultralong GnRHa protocol**		**Antagonist protocol**		
	Park *et al*., 2016^*^	Gosereline 3,75 mg monthly. Dose repeated up to 3 months if AP uterine diameter was ≥ 70mm.	Fresh	Daily dose (0.25mg) of GnRH-ant on iniciated based on a flexible protocol, once a folicle reached ≥ 12mm and until trigger day	Fresh	Clinical pregnancy, miscarriage rate
	Ge *et al*., 2023	Triptoreline acetate 3.75mg monthly. Dose repeated up to 6 months if AP uterine diameter was ≥ 70mm.	Fresh	Daily dose (0.25mg) of GnRH-ant on iniciated based on a flexible protocol, once a folicle reached ≥ 12mm and until trigger day	Fresh	(see above)
		**Long GnRHa protocol**		**Antagonist protocol**		
	Ge *et al*., 2023	Triptoreline acetate (0.05-0.1mg) daily dose for 14 days	Fresh	Daily dose (0.25mg) of GnRH-ant on iniciated based on a flexible protocol, once a folicle reached ≥ 12mm and until trigger day	Fresh	(see above)
		**Short GnRHa protocol**		**Antagonist protocol**		
	Ge *et al*., 2023	Triptoreline acetate (0.05-0.1mg) daily dose on days 2-4 of the menstrual cycleuntil HCG trigger	Fresh	Daily dose (0.25mg) of GnRH-ant on iniciated based on a flexible protocol, once a folicle reached ≥ 12mm and until trigger day	Fresh	(see above)
**Pretreatment before FET**		**Pretreatment with GnRHa**		**No pretreatment**		
	Niu *et al*., 2013^*^	Leuproline acetate 3.75mg for 28 days followed by leuproline acetate 1.875 mg. 21 days after HRT was initiated.	Artificial cycle	No pretreatment before HRT	Artificial cycle	Implantation rate, clinical pregnancy
	Li *et al*, 2021^*^	Triptoreline or leuprolide 3,75mg for 28 days. Patients with larger uterine volumes were administered additional injections.	Artificial cycle	No pretreatment before HRT	Artificial cycle	Implantation, clinical pregnancy, miscarriage and live birth rate
	Zhang *et al*., 2022	Triptoreline acetate 3,75mg monthly for 3 months before HRT. Some patients underwent FET < 3 months pretreatment	Artificial cycle	No pretreatment before HRT	Artificial cycle	Clinical pregnancy rate, live birth rate, miscarriage rate

* No data regarding the protocol for COS is provided the authors.

**Table 2:** Description of the population included by type of intervention and treatment group.

	Study	Study Design	Inclusion criteria	Treatment group				Treatment group				*P*
				N Cycles	Age (years)	Ovarian reserve^*^	Nº ET (n)	N Cycles	Age (years)	Ovarian reserve^*^	Nº ET (n)	
				Ultralong GnRH protocol				Long GnRH protocol				
GnRH protocol before COS and fresh ET	Hou *et al* 2020	Retrospective cohort	Infertile patients whith adenomyosis. Age<38, FSH<10, AFC>10, first IVF attemps, no PCOS, no imune diseases, no myomas, no previous myomectomy, no uterine malformations, no ednometriosis	362	31.9 (30-35)	*6.5* *(5.34-7.58)*	NR	127	31.8 (29-34)	*6.3* *(5.4-7.1)*	NR	NS
	Chen *et al*., 2020	Retrospective cohort	Infertile patients with adenomyosis	52	33.5 (31-36.75)	*6.19* *(4.99-7.39)*	2 (2-3)	162	33.5 (30-36.75)	*5.74* *(4.88-6.74*)	2 (2-3)	NS
	Lan *et al*., 2021	Retrospective cohort	Infertile patients whith adenomyosis. Age<42, no myomas, no previous uterine surgery, no uterine malformations, no untreated hydrosalpinx	237	33.55±4.12	2.37 (1.35-4.17)	2.05±0.57	134	33.99±4.08	1.93 (1.13-4.18)	2.05±0.55	NS
	Wu *et al*., 2022	Retrospective cohort	Infertile patients whith adenomyosis. Age<42, no submucosal myoma or myoma<2cm, no previous uterine surgery, no uterine malfromatios, no untreated hydrosalpix, <3 attemps on ET	259	33.35±4.16	8.09±3.74	2.01±0.54^a^	115	33.88±4.13	*8.80±4.11*	1.95±0.5^a^	0.11^a^
	Ge *et al*., 2023	Retrospective cohort	Infertile patients whith adenomyosis. Age<42, no intrauterine lesions, no uterine malformations, no untreated hydrosalpinx	108	34 (31-37)	1.65 (1.06-3.03)^b^	NR	56	32.5 (30-36)	*2.75* *(1.66-3.90)^b^*	NR	0.11^b^
				**Ultralong GnRH protocol**				**Antagonist GnRH protocol**				
	Park *et al*., 2016	Retrospective cohort	Infertile patients whith adenomyosis, excluding other causes of infertility other than adenomyosis	150	35.2±3.5	*8.2±7.0*	2.9±1.1	147	36.1±3.3	*10.5±2.3*	2.7±1.1	NS
	Ge *et al*., 2023	Retrospective cohort	(see above)	162	34 (31-37)	1.65 (1.06-3.03)	NR	34	33.5 (32-41)	1.32 (0.92-3.37)	NR	NS
				**Long GnRH protocol**				**Antagonist GnRH protocol**				
	Ge *et al*., 2023	Retrospective cohort	(see above)	56	32.5 (32-1)	2.75 (1.66-390)	NR	34	33.5 (32-41)	1.32 (0.92-3.37)	NR	NS
					Short GnRH protocol				Antagonist GnRH protocol			
	Ge *et al*., 2023	Retrospective cohort	(see above)	59	33.5 (32-41)	1.30 (0.77-1.90)	NR	34	33.5 (32-41)	1.32 (0.92-3.37)	NR	NS
**Pretreatment before FET**					**GnRH pretreatment**				**No pretreatment**			
	Niu *et al*., 2013	Retrospective cohort	Infertile patients with adenomyosis, Age <38, ≤ 1 previous FET, normal uterine cavity, no previous myomectomy, no untreated hydrosalpinx, no stage IV endometriosis	194	32.11±4.02	*7.46±2.27*	1.96±.04	145	31.52±4.03	*7.19±1.33*	1.94±0.37	NS
	Zhang *et al*., 2022	Retrospective cohort	Infertile patients with adenomyosis. Age ≤45, uterine volume between 56-100cm^3^, no endocrine or autoimmune disorders, normal kariotype in both partners, normal uterine cavity (no intrauterine adhesions, no submucosal myomas or myomas <50mm)	45	33.5±3.7	3.36±	1.48±0.6	218	34.8±4.7	3.50±2.9	1.49±0.6	NS
	Li *et al*., 2021	Retrospective cohort	Infertile patients with adenomyosis. Age <45, no uterine malformations, no endometriosis, no untreated hydrosalpinx, no PCOS, normal kariotype in both partners	160	35.56±4.49	*6.54±1.89*	1.11±0.19	181	35.25±4.95	*6.32±2.04*	1.12±0.21	NS

* Ovarian reserve: AMH expressed in ng/ml or basal FSH in italics and expressed in mlU/ml when AMH was not available; PCOS = Polycystic Ovarian Syndrome.

### Assessment of risk of bias

**Figure 1:**
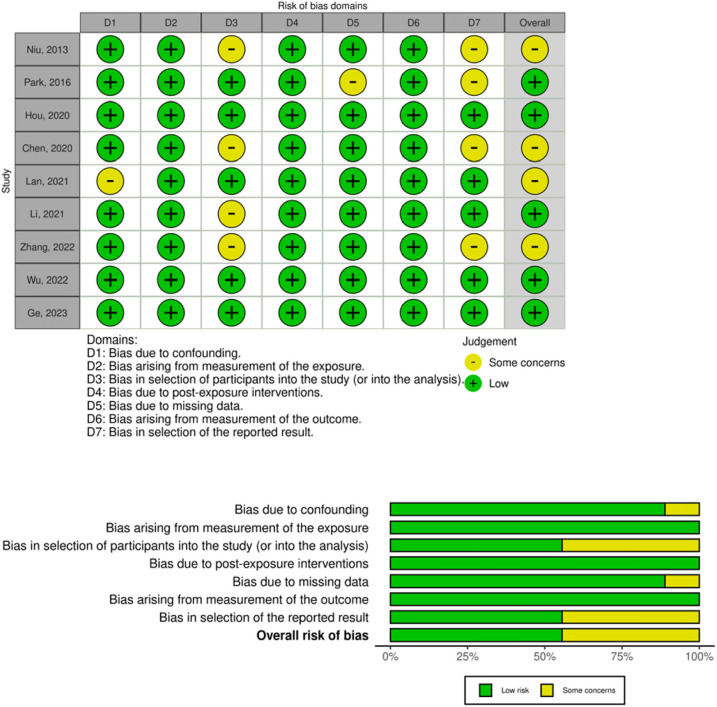
Assessment of the quality of the included studies (ROBINS-E).

We assessed the risk of bias in the included studies following the guidance suggested by the Cochrane Collaboration for non-randomized studies, using ROBINS-E tool (Higgins *et al.*, 2024). We addressed seven specific domains, which include i) confounding, ii) measurement of exposure, iii) selection of participants into the study, iv) Post-exposure interventions, v) Missing data, vi) measurement of the outcome, and vii) selection of the reported results. A judgment of “Low” for all domains indicates a low risk of bias, a judgment of “High” for one or more domains indicates a high risk of bias. We interpreted the risk of bias in the specific domains as “Some concerns” when the information was unclear. The risk of bias for the included trials is detailed in Fig. 1.

### Statistical analysis

Statistical analyses were performed using Review Manager software (version 5.3; Copenhagen: Nordic Cochrane Centre, Cochrane Collaboration, 2014). The odds ratio (OR) and 95 confidence interval (CI) was used for the outcomes described. The degree of heterogeneity was evaluated through I2 statistic, and tests for subgroup differences were based on random-effects models in order to minimize the false-positive results (Higgins *et al.*, 2003). Thresholds for the interpretation of I^2^ is as follows: 0-40% might not be important, 30-60% may represent moderate heterogeneity, 50-70% may represent substantial heterogeneity, and 75% to 100% considerable heterogeneity. From 30% to 100%, the importance of the I^2^ value will depend on the magnitude and direction of the effect as well as the strength of evidence (*p* value <0.10 shows statistical significance) (Deeks *et al.*, 2024).

## RESULTS

**Figure 2:**
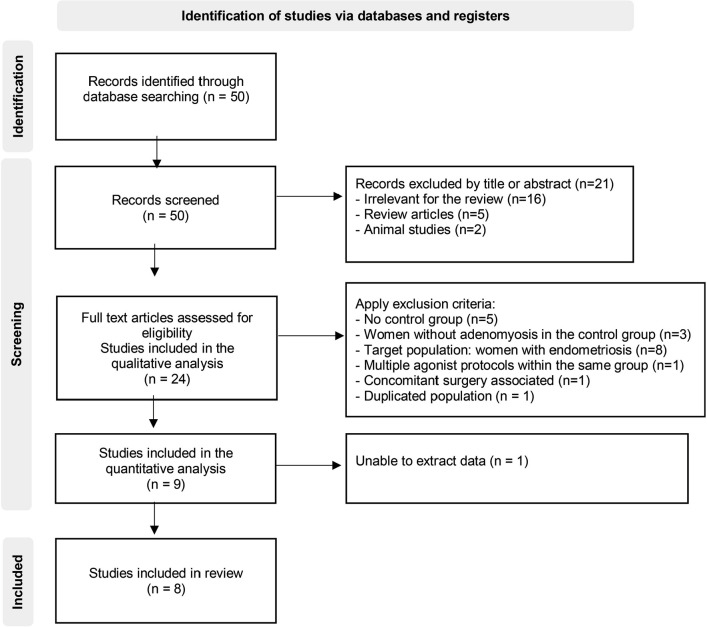
Flow chart for the trial identification and selection process.

A total of 50 articles were identified in the initial electronic search, but only 24 were considered for eligibility. During the second phase of the inclusion process, 15 studies were excluded because they had no control group for comparison (n=5), the control group was comprised of women without endometriosis (n=3), the target population of the study was women with endometriosis and concomitant adenomyosis (n=8), combined multiple agonist protocols within the same group (n=1) or combined surgery with the agonist protocol (n=1). Two studies were excluded because of i) a potential duplicate (Li *et al.*, 2023) since it included women possibly evaluated in a separate cohort study (Ge *et al.*, 2023), or ii) were unable to extract data (even though the authors had been contacted directly (Dian *et al.*, 2022). After screening, 8 cohort studies were finally included in the metaanalysis. The article search and screening process is shown in Fig. 2. The two reviewers achieved good agreement on the selection of the trials (weighted kappa 0.75).

### Description of included studies

Eight retrospective cohort studies were included. They were grouped according to the comparison in i) ultralong versus long GnRHa protocol before COS and fresh ET (Chen *et al.*, 2020; Hou *et al.*, 2020; Lan *et al.*, 2021; Wu *et al.*, 2022; Ge *et al.*, 2023), ii) ultra-long GnRHa versus antagonist protocol before COS and fresh ET (Park *et al.*, 2016; Ge *et al.*, 2023), iii) long GnRHa versus antagonist protocol before COS and fresh ET (Ge *et al.*, 2023), iv) short GnRHa versus antagonist protocol before COS and fresh ET (Ge *et al.*, 2023), v) Long GnRH agonist pretreatment versus no treatment before FET regardless of the protocol followed for COS (Niu *et al.*, 2013; Li *et al.*, 2022; Zhang *et al.*, 2022).

The mean age across the studies was comparable. The inclusion criteria were relatively homogeneous. Most studies included women under the age of 42-45 years, although the studies by Hou *et al.* (2020) and Niu *et al.* (2013)only considered for inclusion women under 38 years. Park *et al.* (2016) did not consider age an inclusion criteria. The ultrasonographic criteria for diagnosing adenomyosis was also relatively homogeneous across the studies, although the group of Zhang failed to provide information regarding this aspect (Zhang *et al.*, 2022).

### Ultra-long versus long GnRH agonist protocol before COS and fresh ET

Five retrospective cohort studies were identified that reported on reproductive outcomes after ultralong and long GnRH agonist protocol before COS and fresh ET (Chen *et al.*, 2020; Hou *et al.*, 2020; Lan *et al.*, 2021; Wu *et al.*, 2022; Ge *et al.*, 2023). A total of 1018 cycles were included in the ultralong GnRH agonist protocol and 594 in the long GnRH agonist protocol.

The inclusion and exclusion criteria were relatively homogeneous across the studies (table 2). Only women under 42 years were included, without uterine malformations, submucosal myomas or untreated hydrosalpinx (Hou *et al.*, 2020; Lan *et al.*, 2021; Wu *et al.*, 2022; Ge *et al.*, 2023), although the study by Hou *et al.* (2020) had more restrictive criteria and only considered women under 38 and with good ovarian reserve (basal FSH<10 mU/mL, and AFC above 10). In the study by Chen *et al.* (2020) exclusion criteria were not reported.

Concerning the basal characteristics, women who received ultralong GnRHa protocol before COS had overall larger uterine size compared to long GnRHa protocol (Lan *et al.*, 2021; Wu *et al.*, 2022; Ge *et al.*, 2023), although in the study by Chen *et al.* (2020) no differences were registered in this regard, and (Hou *et al.*, 2020) did not report on this variable. However the studies that registered the type of adenomyosis of women included (focal versus diffuse) reported no differences between treatment groups (Lan *et al.*, 2021; Wu *et al.*, 2022).

Regarding ovarian reserve status of women included, measured by AMH (or AFC and basal FSH when AMH was no available), 4 studies reported no differences between groups (Chen *et al.*, 2020; Hou *et al.*, 2020; Lan *et al.*, 2021; Wu *et al.*, 2022). In the study by Ge *et al.* (2023), a significantly lower AMH was registered among women in the ultralong GnRHa group (*p*<0.001).

Additionally, in terms of coexisting of endometriosis, a significantly higher percentage of endometriosis (Lan *et al.*, 2021) or moderate/severe dysmenorrhea (Ge *et al.*, 2023) was reported among women in the ultralong GnRHa protocol compared to the long GnRH protocol. In the study by Wu *et al.* (2022), both groups were comparable, although Chen *et al.* (2020) did not report on this variable. The group of Hou *et al.* (2020) considered endometriosis as an exclusion criteria (Table 2).

With regard to the protocols reported before COS, most authors reported ultra-long treatment with GnRH analogues over 2 to 4 months (Chen *et al.*, 2020; Hou *et al.*, 2020; Lan *et al.*, 2021; Wu *et al.*, 2022), although Ge included women who received only one month but could extend the treatment up to 6 months depending on the uterine diameter (Ge *et al.*, 2023). The long GnRHa protocol was more comparable across studies, starting 10 to 14 days before the beginning of the COS (Table 1).

### Ultra-long GnRHa versus antagonist protocol before COS and fresh ET

Two retrospective studies evaluated reproductive outcomes between women who received ultra-long GnRHa protocol compared antagonist protocol before COS and fresh ET (Park *et al.*, 2016; Ge *et al.*, 2023). A total of 267 cycles were evaluated in the group of ultra-long GnRHa protocol and 181 cycles in the antagonist protocol (Table 2).

Regarding basal characteristics, both groups of treatment across the studies were comparable in terms of ovarian reserve. However, (Ge *et al.*, 2023) registered a significantly higher history of severe dysmenorrhea in the ultralong GnRHa protocol (37.96% versus 11.76%, *p*<0.001).

None of the studies registered uterine volumes or type of adenomyosis between groups, and Park *et al.* (2016) did not report on previous history of either dysmenorrhea or coexisting endometriosis.

### Long GnRHa versus antagonist protocol before COS and fresh ET

Only one retrospective study evaluated reproductive outcomes between women who received long GnRHa protocol before COS and fresh ET compared antagonist protocol (Ge *et al.*, 2023). A total of 56 cycles of women who received long GnRHa protocol compared to 34 with antagonist protocol for COS and fresh ET were included (Table 2).

Regarding basal characteristics, women who received long GnRHa had significantly smaller uterine size and better ovarian reserve compared to women in the antagonist protocol, and no differences in the previous history of dysmenorrhea we registered.

### Short GnRHa versus antagonist protocol before COS and fresh ET

Only one retrospective study evaluated reproductive outcomes between women undergoing short GnRHa compared to antagonist protocol before COS and fresh ET, with a total of 59 and 34 cycles included, respectively (Ge *et al.*, 2023).

No differences were registered regarding ovarian reserve or previous history of dysmenorrhea. However, no information was given regarding the uterine volume or the type of adenomyosis.

### Pretreatment with GnRHa versus no pretreatment before FET

Three retrospective studies evaluated the use of the pretreatment with GnRHa compared with no pretreatment before FET (Niu *et al.*, 2013; Li *et al.*, 2022; Zhang *et al.*, 2022). In all, 399 cycles of women with adenomyosis who received at least 28 days of GnRH pretreatment before FET were included, while 544 underwent FET without pretreatment (Table 2).

Regarding the basal characteristics, ovarian reserve was comparable between groups of treatment in all studies. Regarding the inclusion of women with endometriosis, only Niu *et al.* (2013) reported on this variable, and described no differences between groups (*p*=0.18).

Regarding the sonographic features of adenomyosis, Zhang *et al.* (2022) Zhang only considered for inclusion women with uterine volumes between 56 and 100cm^3^. In this study, both groups were balanced regarding the size of the uterus (p=0.220), and the presence of focal versus diffuse adenomyosis (*p*=0.145). However, neither the group of Niu nor Li reported on these variables (Niu *et al.*, 2013; Li *et al.*, 2022).

The three studies failed to provide data regarding the stimulation protocol used for COS before embryo cryopreservation. With regard to the pretreatment received before FET, Niu *et al.* (2013) and Li *et al.* (2022) used GnRHa during 28 days, while Zhang *et al.* (2022) extended the treatment up to 3 months. Regarding the group who received no pretreatment, Niu *et al.* (2013) and Li *et al.* (2022) used hormonal replacement therapy before FET, while Zhang *et al.* (2022) used the natural cycle for endometrial preparation (Table 1).

### Internal validity of included studies

In general, studies provided data regarding methodological aspects particularly with reference to the inclusion and exclusion criteria, the mode of diagnosis of the disease and the protocols used for either controlled ovarian stimulation or the treatment received before FET, depending on the study.

As shown in Figure 1, and according to the ROBINS-E tool (Higgins *et al.*, 2024), studies showed in general low risk of bias in terms of bias due to confounding, bias arising from measurement of the exposure, bias due to post-exposure interventions, due to missing data and bias in measurement of the outcome.

However, in four of the included studies, the authors (Niu *et al.*, 2013; Chen *et al.*, 2020; Li *et al.*, 2021; Zhang *et al.*, 2022) failed to provide in detail some characteristics regarding the population included that would allow comparison across studies, especially regarding the co-existence of endometriosis and the type of adenomyosis, raising some concerns regarding the risk of bias in selection of participants into the study.

In addition, there were some concerns regarding the selection of the reported results, and some authors failed to provide data for some of the main outcomes of interest (Niu *et al.*, 2013; Park *et al.*, 2016; Chen *et al.*, 2020; Zhang *et al.*, 2022).

### Outcomes of interest

#### Ultra-long versus long GnRH agonist protocol before COS and fresh ET

**Figure 3:**
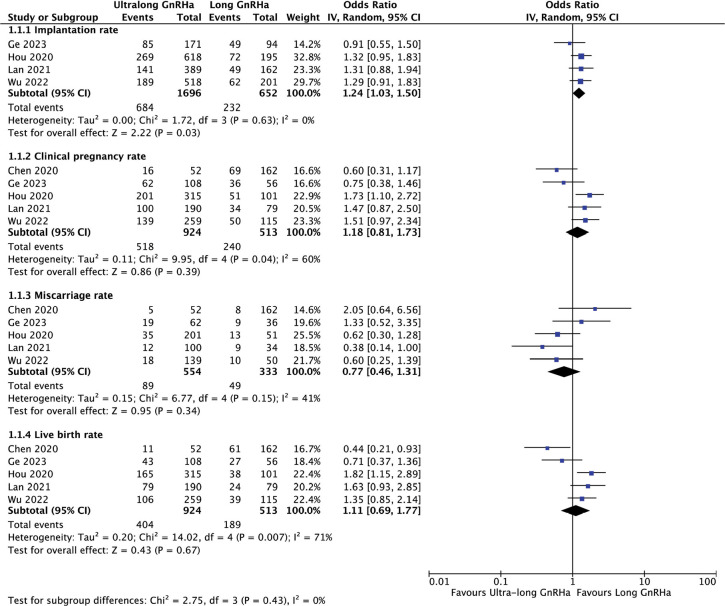
Reproductive outcomes after Ultra-long compared to Long GnRHa protocol for COS and fresh ET.

The pooled analysis of the data from the 5 studies (Figure 3) showed no significant differences in live birth (OR 1.1, 95% CI 0.69-1.77), clinical pregnancy (OR 0.77, 95% CI 0.46-1.31) or miscarriage rates (OR 1.18, 95% CI 0.81-1.73) when comparing the use of ultra-long versus long GnRHa protocols for COS and fresh ET, However, a significant increase in implantation rate was detected (OR 1.24, 95% CI, 1.03-1.,50), although only 4 of the studies reported on this outcome (Hou *et al.*, 2020; Lan *et al.*, 2021; Wu *et al.*, 2022; Ge *et al.*, 2023). The test for heterogeneity across subgroup showed low heterogeneity (I^2^=0%).

#### Ultra-long GnRHa versus antagonist protocol before COS and fresh ET

**Figure 4:**
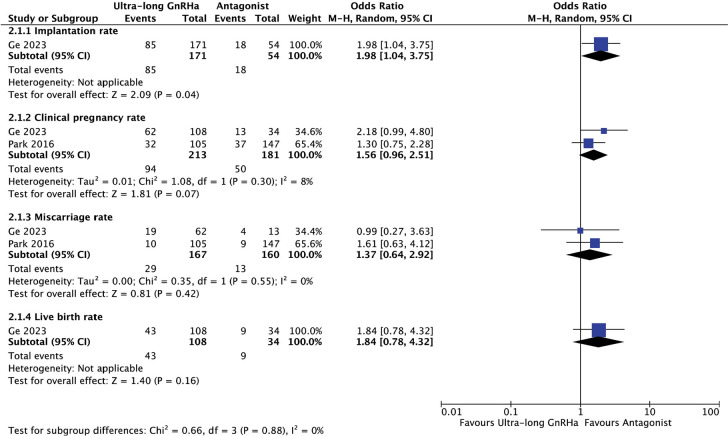
Reproductive outcomes after Ultra-long GnRHa compared to Antagonist protocol for COS and fresh ET.

With regard to the use of ultra-long GnRHa versus antagonist protocols for COS (Figure 4), the pooled analysis of the data showed no differences in live birth (OR 1.84, 95% CI 0.78-4.32), clinical pregnancy (OR 1.56, 95% CI 0.96-2-51) or miscarriage rates (OR 1.37, 95% CI 0.64-2.92). In contrast, a significant increase in implantation rate was observed (OR 1.98, 95% CI 1.04-3.75). However, only the study by Ge *et al.* (2023) reported on both implantation and live birth rates.

The test for heterogeneity across subgroup showed low heterogeneity (I^2^=0%).

#### Long GnRHa versus antagonist protocol before COS and fresh ET

**Figure 5:**
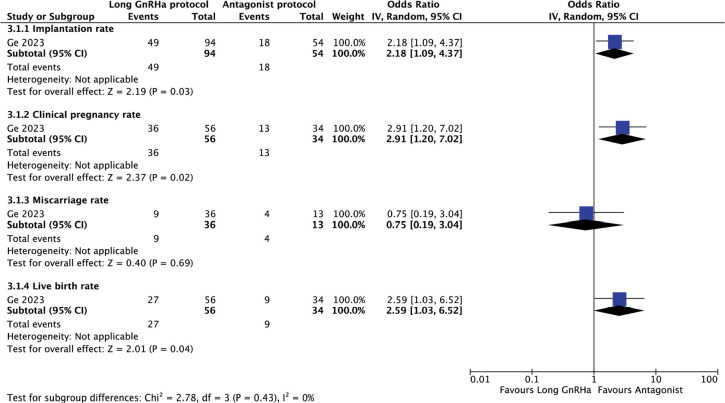
Reproductive outcomes after Long GnRHa compared to Antagonist protocol for COS and fresh ET.

The use of long GnRHa compared with the antagonist protocol, only one study reported this comparison (Ge *et al.*, 2023). A significant increase in live birth (OR 2.59, 95% CI 1.03-6.52), clinical pregnancy (OR 2.91, 95% CI 1.20-7.02) and implantation rates (OR 2.18, 95% CI 1.09-4.37) were observed (Figure 5). However, no differences we observed in the miscarriage rate (OR 0.75, 95% CI 0.19-3.04).

The test for heterogeneity across subgroup showed low heterogeneity (I^2^=0%).

#### Short GnRHa versus antagonist protocol before COS and fresh ET

**Figure 6:**
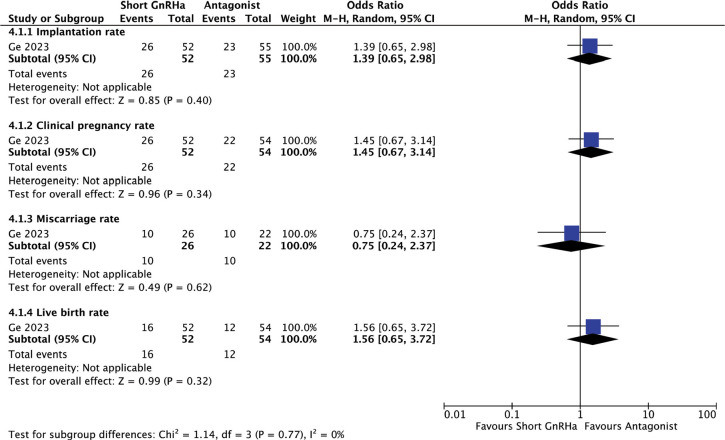
Reproductive outcomes after Short GnRHa compared to Antagonist protocol for COS and fresh ET.

Only one study compared the use of short GnRH versus antagonist protocol for COS (Ge *et al.*, 2023). The analysis showed no significant differences in terms of live birth, clinical pregnancy, miscarriage or implantation rates (Figure 6).

The test for heterogeneity across subgroup showed low heterogeneity (I^2^=0%).

#### Pretreatment with GnRHa versus no pretreatment before FET

**Figure 7:**
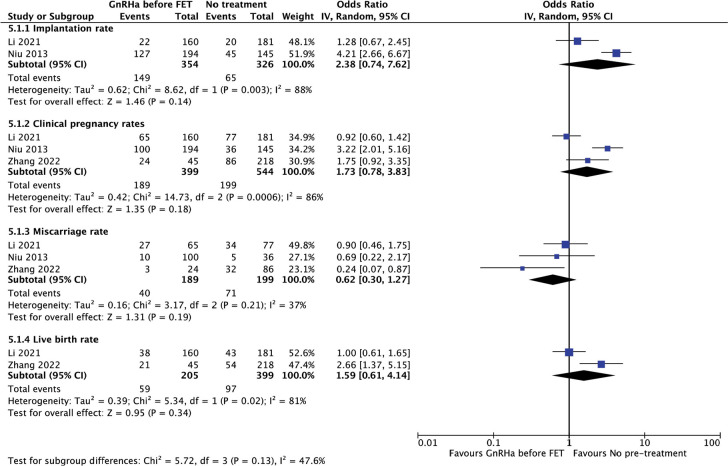
Reproductive outcomes after pretreatment with GnRHa before FET versus no pretreatment.

With regarding to the use of GnRH analogues as a pretreatment before FET compared to no treatment, no significant differences were registered in terms of live birth, clinical pregnancy, miscarriage or implantation rates in the pooled analysis of the data (Figure 7).

Only two of the three studies provided data regarding implantation (Niu *et al.*, 2013; Li *et al.*, 2022) and live birth rates (Li *et al.*, 2022; Zhang *et al.*, 2022).

The test for heterogeneity across subgroup showed moderate heterogeneity (I^2^=47.6%), value considered non significant (*p*=0.13).

## DISCUSSION

This systematic review and meta-analysis show that pituitary downregulation before controlled ovarian stimulation with either long or ultra-long protocols among women with adenomyosis does not improve reproductive outcomes. In fact, the use of ultra-long GnRHa protocols compared to either long GnRHa or antagonist protocol seem to significantly lower implantation rate, although results in terms of live birth rate were comparable. However, the use of long GnRHa protocol before COS seem to negative affect reproductive outcomes compared to antagonist protocols, including implantation, clinical pregnancy, live birth rates.In addition, the pretreatment with GnRHa before FET compared to no treatment does not offer any significant benefit in reproductive outcomes among these women.

During the past decade, several authors have described how adenomyosis is associated with poorer reproductive outcomes compared to women without the disease (Vercellini *et al.*, 2014; Younes and Tulandi, 2017; Horton *et al.*, 2019; Bourdon *et al.*, 2021; Nirgianakis *et al.*, 2021; Cozzolino *et al.*, 2022). However there has been high heterogeneity within and across groups that limits the internal and external validity of the results observed. Studies that evaluated IVF results among women with and without adenomyosis were based on women with colorectal endometriosis (Ballester *et al.*, 2012), used oocyte donated cycles (Martínez-Conejero *et al.*, 2011), or used the junctional zone thickness (Maubon *et al.*, 2010) or even the myometrial thickness (Youm *et al.*, 2011) as an indirect marker to define women with adenomyosis.

In this regard, the use of GnRHa have been extensively used for the treatment of adenomyosis, offering a reduction in uterine volume and also a relief in pain symptoms (Grow & Filer, 1991; Nelson & Corson, 1993; Akira *et al.*, 2009). However, their potential beneficial effect of IVF treatments is yet to be established. So far, only two meta-analysis included studies that evaluated the different IVF treatments among women with adenomyosis (Younes and Tulandi, 2017; Cozzolino *et al.*, 2022). Yet, a comparison between treatments could not be made, since different protocols were used. The meta-analysis by Younes & Tulandi (2017) reported that the use of GnRHa protocols for COS could be beneficial to improve IVF outcomes in adenomyosis, although they only included the study by Niu *et al.* (2013), that assessed GnRHa pre-treatment compared with no treatment before FET, and the study by Park *et al.* (2016) that compared long versus antagonist GnRH protocols for COS. The meta-analysis by Cozzolino *et al.* (2022) additionally included a third study (Hou *et al.*, 2020) that assessed the use of ultra-long versus long GnRHa before COS and fresh ET, and authors concluded that with available evidence, the use of long-term GnRHa did not seem beneficial.

This latter meta-analysis also included the study by Zhang *et al.* (2022), however the goal of this one was to evaluate whether the use of surgery among women undergoing IVF with GnRHa protocols could be beneficial among these women. Interestingly, several authors have focused on evaluating the different adenomyosis phenotypes to better understand their impact on fertility (Bourdon *et al.*, 2020; Cozzolino *et al.*, 2024).In fact, the inner and outer myometrium are two functionally different entities (Chapron *et al.*, 2017; Khan *et al.*, 2019; Bourdon *et al.*, 2020). In this regard, Cozzolino *et al.* (2024) reported in a prospective study a threefold higher relative risk of miscarriage when adenomyosis involved the inner myometrium, in contact with the Junctional zone, and a higher ongoing pregnancy rate when adenomyosis was exclusively in the outer myometrium.

Not only the location, but also the type of adenomyosis has been the spotlight among infertile women with adenomyosis. Han *et al.* (2023) compared retrospectively the use of ultra-long GnRHa protocol prior to COS and reported worse reproductive outcomes among women with diffuse adenomyosis in terms of clinical pregnancy, miscarriage and live birth compared with focal adenomyosis, although this latter group still showed worse miscarriage rates compared with women with tubal infertility.

In this regard, the study by Lan *et al.* (2021) included in the present meta-analysis, reported no significant differences between the use of ultra-long versus long GnRHa protocols before COS, except for a reduction in early miscarriage rate in the group who received ultra-long GnRHa (*p*=0.045). Interestingly, when stratifying by the type of adenomyosis, women with diffuse adenomyosis showed an increase in clinical pregnancy and live birth rates when using ultra-long GnRHa compared with long protocols, while the group with focal adenomyosis showed comparable results. Other authors have focused on the uterine volume, as an indicator of severity, since it reveals the accumulation of adenomyotic tissue within the myometrium (Li *et al.*, 2021; Han *et al.*, 2023; Cozzolino *et al.*, 2024). In this matter, Cozzolino *et al.* (2024) revealed worse reproductive outcomes in severe cases of adenomyosis, while Zhang *et al.* (2022) described turning point of uterine volumes larger than 8 weeks gestation (130cm^3^) for worse reproductive outcomes in terms of miscarriage and live birth rates (Han *et al.*, 2023).

Among the studies included in this meta-analysis, several authors provided details regarding the uterine size of the study population (Chen *et al.*, 2020; Lan *et al.*, 2021; Wu *et al.*, 2022; Zhang *et al.*, 2022; Ge *et al.*, 2023). Only in three of them a significantly larger uterine size was registered in the group who received ultra-long GnRHa compared to those in the long GnRHa group. Yet none of them performed subanalysis of the reproductive outcomes according to the uterine volume or even reported the uterine size reduction after the treatment with ultra-long GnRHa treatment before initiating COS.

So far, even though adenomyosis has been associated to negative IVF outcomes (Vercellini *et al.*, 2014; Younes and Tulandi, 2017; Horton *et al.*, 2019; Huang *et al.*, 2020; Nirgianakis *et al.*, 2021; Cozzolino *et al.*, 2022; Moawad *et al.*, 2022), there is lack of evidence regarding the benefits of long or ultra-long GnRHa protocols in reproductive outcomes among these women. In addition, even though there is some evidence that different types of adenomyosis or even the uterine volume could have different negative effect of reproductive outcomes, the potential benefit of these protocols for COS and ET is yet to be addressed.

Certainly, there are cases where the GnRHa-induced hypopituarism has failed to reduce the estrogen production within adenomyotic tissue particularly in severe cases (Cozzolino *et al.*, 2023), leading to consider adjuvant treatments. The increased aromatase activity in adenomyotic tissue (Kitawaki *et al.*, 1997) has led to the use of aromatase inhibitors (AI) in symptomatic women with adenomyosis undergoing IVF (Kimura *et al.*, 2007; Badawy *et al.*, 2012; Sharma *et al.*, 2023). Interestingly, Sharma *et al.* (2023) reported in a randomized controlled trial that the use of low-dose letrozole could be an effective option for women with symptomatic adenomyosis awaiting IVF. In addition, AI adjuvant to FSH treatment seem to offer IVF outcomes comparable to standard IVF, reaching lower estradiol concentrations in blood (Requena *et al.*, 2008; Lin *et al.*, 2024). Unfortunately, there is lack of research comparing different IVF protocols among women with adenomyosis undergoing pre-treatment with AI prior to ovarian stimulation, or even comparing different IVF protocols using AI adjuvant to FSH during ovarian stimulation.

### Limitations

This meta-analysis has several potential limitations that need to be pointed out. First, the retrospective nature of all studies included could compromise the homogeneity of women who received different IVF protocols and affect negatively on reproductive outcomes regardless of the treatment received. In fact, women who received ultra-long GnRH protocol had larger uterine volumes (Lan *et al.*, 2021; Wu *et al.*, 2022; Ge *et al.*, 2023) and in some studies a higher rate of dismenorrea (Ge *et al.*, 2023) or confirmed endometriosis (Lan *et al.*, 2021), and even lower AMH levels compared with long GnRHa protocol (Ge *et al.*, 2023). Unfortunately not all studies provided with this data, limiting the interpretation of the results from this meta-analysis. Additionally, none of the studies that evaluated the use of pre-treatment with GnRH before FET reported on the type of protocol used for COS.

Although unavoidable, the studies included in our meta-analysis are heterogeneous, which could lead to some biases in the results. Particularly, the duration of the ultra-long GnRH treatments received, since it varies across studies, but also within each study. In some studies, the length of the treatment depended on the reduction of the each patient’s uterine size (Park *et al.*, 2016; Chen *et al.*, 2020; Wu *et al.*, 2022; Ge *et al.*, 2023).

### Strengths

This is the first meta-analysis to compare the different IVF protocols among women with adenomyosis. Studies that included women with endometriosis and concomitant adenomyosis, that combined other strategies to the IVF treatment such as surgery, or mixed different protocols within the same group were excluded, in order to minimize bias arising from measurement of the exposure or due to post-exposure interventions (Wang *et al.*, 2009; Mijatovic *et al.*, 2010; Al Jama, 2011; Ballester *et al.*, 2012). Nonetheless, we need to identify what types of adenomyosis are associated with poorer reproductive outcomes.

## CONCLUSIONS

There is no evidence regarding the benefit of pituitary downregulation before controlled ovarian stimulation or even as pre-treatment before frozen embryo transfer systematically among women with adenomyosis undergoing IVF. In fact, the use of long GnRHa seem to worsen reproductive outcomes compared to antagonist protocols. Additionally, the pretreatment with GnRHa before FET among women with adenomyosis show not beneficial effect.

So far, there is lack of evidence regarding the benefits of the pituitary downregulation among women with specific subtypes of adenomyosis that entail worse reproductive prognosis, such as adenomyosis of the inner myometrium or even women with larger uterine volumes. Results from this systematic review and meta-analysis should raise awareness of the risks of using pituitary downregulation systematically among women with adenomyosis undergoing IVF. There is a need for prospective trials that assess the benefits of GnRH analogues before COS or as pretreatment before FET among women with different types of adenomyosis.
